# Use of Virtual Reality to Assess Dynamic Posturography and Sensory Organization: Instrument Validation Study

**DOI:** 10.2196/19580

**Published:** 2020-12-16

**Authors:** Matthew William Wittstein, Anthony Crider, Samantha Mastrocola, Mariana Guerena Gonzalez

**Affiliations:** 1 Department of Exercise Science Elon University Elon, NC United States; 2 Department of Physics Elon University Elon, NC United States

**Keywords:** postural control, virtual reality, sensory organization test, intraclass correlations

## Abstract

**Background:**

The Equitest system (Neurocom) is a computerized dynamic posturography device used by health care providers and clinical researchers to safely test an individual’s postural control. While the Equitest system has evaluative and rehabilitative value, it may be limited owing to its cost, lack of portability, and reliance on only sagittal plane movements. Virtual reality (VR) provides an opportunity to reduce these limitations by providing more mobile and cost-effective tools while also observing a wider array of postural characteristics.

**Objective:**

This study aimed to test the plausibility of using VR as a feasible alternative to the Equitest system for conducting a sensory organization test.

**Methods:**

A convenience sample of 20 college-aged healthy individuals participated in the study. Participants completed the sensory organization test using the Equitest system as well as using a VR environment while standing atop a force plate (Bertec Inc). The Equitest system measures the equilibrium index. During VR trials, the estimated equilibrium index, 95% ellipse area, path length, and anterior-posterior detrended fluctuation analysis scaling exponent alpha were calculated from center of pressure data. Pearson correlation coefficients were used to assess the relationship between the equilibrium index and center of pressure–derived balance measures. Intraclass correlations for absolute agreement and consistency were calculated to compare the equilibrium index and estimated equilibrium index.

**Results:**

Intraclass correlations demonstrated moderate consistency and absolute agreement (0.5 < intraclass correlation coefficient < 0.75) between the equilibrium index and estimated equilibrium index from the Equitest and VR sensory organization test (SOT), respectively, in four of six tested conditions. Additionally, weak to moderate correlations between force plate measurements and the equilibrium index were noted in several of the conditions.

**Conclusions:**

This research demonstrated the plausibility of using VR as an alternative method to conduct the SOT. Ongoing development and testing of virtual environments are necessary before employing the technology as a replacement to current clinical tests.

## Introduction

The Equitest system (Neurocom) is a computerized dynamic posturography device used by health care providers and clinical researchers to safely test an individual’s postural control. Implementing the sensory organization test (SOT) using the Equitest system requires individuals to process and integrate cues from the visual, vestibular, and proprioceptive systems. This test provides clinicians and researchers with an equilibrium score for each tested condition, a sensory analysis score, a strategy analysis, and a center of gravity (COG) alignment. While the Equitest system has evaluative and rehabilitative value, it may be limited owing to its cost and lack of portability. Moreover, the performance variables provided by the Equitest system are limited, representing gross outcome measures derived only from sagittal plane movement dynamics [1]. Recent advances in technology provide opportunities to reduce these limitations by providing more mobile and cost-effective tools while also observing a wider array of postural characteristics. The purpose of this research was to evaluate the validity of using virtual reality (VR) and a force plate as an alternative to the Equitest system.

The SOT has been the dominant clinical test to assess sensory integration in the context of postural control for neurologic disorders and deficits. With the wide use of clinical dynamic posturography over the last 30 years, the Equitest system has become widely accepted as the gold standard to assess postural stability and balance in several populations (eg, children, aging adults, and military personnel) and clinical groups (eg, those with concussion, vertigo, Parkinson disease, and Alzheimer disease). By systematically disrupting the visual and somatosensory information available to an individual, it is possible to distinguish someone’s reliance on the following three major sensory systems during balance tasks: the visual, somatosensory, and vestibular systems. Conveniently, the Equitest system provides an equilibrium score (indicating how little participants swayed) during each test, as well as a sensory analysis score (indicating how much they relied on each system) and strategy analysis (indicating the hip versus ankle strategy) for the battery of conditions.

While the Equitest system provides a quick evaluative tool for clinicians and researchers, it is not without limitations. First, these outcome measures are derived solely from sagittal plane movements and may not reflect a complete assessment of an individual’s postural control. Second, the costs associated with the Equitest system may limit its availability in underserved communities or during times immediately following an injury (such as a sports concussion). As an alternative to the Equitest system, it may be possible to combine more recent technologies, that is, portable force plates and VR, to ameliorate these drawbacks. When these technologies are combined, they greatly reduce the cost for a clinician to own testing equipment, as well as offer the opportunity to have a portable solution that could be taken into the field. Moreover, portable force plates present the possibility to record and assess a wider range of data, such as medial-lateral dynamics, and customize the outcomes to specific clinical goals. Likewise, VR headsets have continued to improve in quality and decrease in cost, and continued developments may lead to the ability to accurately track movements in VR without additional hardware components such as force plates.

In keeping up with technological advancements, it is important to determine how new technologies can measure up to the “gold standards” they will eventually replace. Currently, VR is approaching this standard and is consistently shown to be a valuable tool to conduct postural and motor control research. Previous research has found no difference between static balance in a physical environment versus a virtual environment [2]. Additionally, several scholars have supported the efficacy of VR for use in balance assessments in a range of clinical populations, such as those with concussion, stroke, Parkinson disease, and high age [3-7]. Continuing in this trend, a large body of research has shown positive results in using VR to enhance training and rehabilitation for balance-related dysfunction [8-11]. Overall, VR has been demonstrated to accurately assess balance in addition to providing a customizable means to enhance clinical outcomes.

The purpose of this research was to compare the Equitest system to a VR balance assessment designed to mimic the SOT in a young healthy population. It was hypothesized that the equilibrium score would demonstrate high limits of agreement between the two testing conditions, supporting VR as a viable option to decrease cost and increase the accessibility of postural assessment techniques. By illustrating the viability of VR to emulate current clinical practices, future progress can focus on improving and optimizing the implementation of VR in clinical standards of care and applications to more populations of interest.

## Methods

### Participants

A convenience sample of 20 college-aged individuals (Table 1) was recruited to participate in this study. All participants were healthy individuals with no prior history of neurological or physical injury or dysfunction. Upon arrival, participants provided informed consent. All procedures were approved by the institutional review board, and no adverse events were encountered.

**Table 1:** Participant demographics.

Characteristic	Male (n=7), mean (SE)	Female (n=13), mean (SE)
Age (years)	20.8 (0.4)	20.9 (0.37)
Height (m)	1.79 (0.03)	1.66 (0.02)
Weight (kg)	77.4 (5.58)	62.8 (3.33)

### Experimental Design

After providing informed consent, participants completed a SOT in two blocks, using the Equitest system and using VR. Blocks of tests were counterbalanced, and conditions within blocks were randomized.

During the Equitest SOT, participants wore a harness that supported their weight in case they lost balance. Researchers helped participants into the harness so it fit comfortably and safely. The conditions during the clinical test included (1) eyes open on a stable surface, (2) eyes closed on a stable surface, (3) eyes open with a sway-referenced surround, (4) eyes open on a sway-referenced surface, (5) eyes closed on a sway-referenced surface, and (6) eyes open with both a sway-referenced surround and surface.

**Figure 1:**
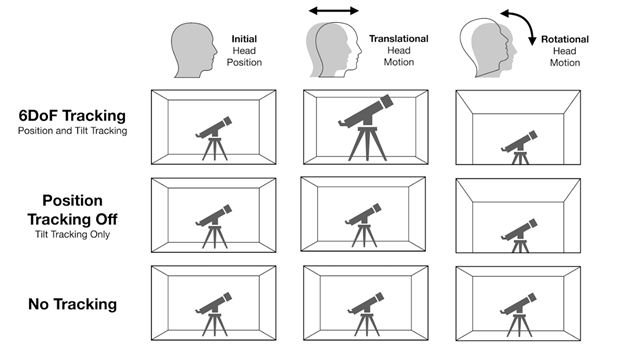
Effect of the head tracking condition in virtual reality on a user's view with translation or rotation of the head. 6DoF: six degrees of freedom.

In the VR SOT, participants removed any glasses and wore a head-mounted display (HTC Vive, HTC). Participants adjusted the headset to ensure clarity in the virtual environment using a black screen with a textbox. To compare our VR SOT to existing SOT research performed with real machines, we created a virtual scale model of the patterns used inside of the Equitest balance system. We placed this model in the center of a white virtual testing room (10 m × 9 m in size). These models and the testing software were created using Unity 3D (v. 2018.2.10f1; Unity Technologies). Our software allowed us to test users with the following three different types of VR tracking: no tracking, head rotation tracking only, and six degrees of freedom (6DoF) tracking (Figure 1). The “no tracking” option creates an experience where the objects viewed move with the user’s head as if they are attached. The second option, which is common in first-generation VR headsets, such as the Oculus DK1 and Google Daydream, is somewhat natural until users lean in a direction that moves their torso. The last of these most closely mimics reality.

Balance was tested in the following conditions in the VR environment: in a completely dark environment, eyes open in an environment that mimics the clinical test (6DoF tracking), eyes open in an environment that mimics the surround of the clinical test and moves and rotates with the participant’s head (no tracking), and eyes open in an environment that mimics the surround of the clinical test and moves forward and backward with the participant’s head but does not react to head rotation (head tracking only). Each condition was completed on a stable surface and on a foam surface.

For each balance condition, in both the clinical test and the VR test, participants completed two trials of 20 seconds. The order of the trials was counterbalanced between the clinical test and VR blocks, and the order of the conditions was randomized within the clinical test and VR blocks. In total, participants completed 28 trials (six clinical testing conditions × two trials each, four VR testing conditions × two surface conditions × two trials each) of 20 seconds of stationary balance. All participants provided written consent prior to beginning the experimental protocol.

### Data Reduction

The Equitest system calculated the equilibrium index (EI) during each SOT condition [12], and it represents the extent to which a participant sways forward or backward within a theoretical limit of 12.5° of displacement. If the participant has no sway, a score of 100 would be received, and if the participant exhibits 12.5° or greater sway (combined forward and backward), a score of 0 would be received. During the VR conditions, participants completed the test on top of a portable force plate (Bertec Inc) that collected center of pressure (COP) data at 50 Hz. Custom MATLAB (Mathworks Inc) scripts were used to detrend and filter the data (20-ms moving average filter) and subsequently calculate the estimated equilibrium index (eEI), 95% ellipse area, path length, and anterior-posterior (AP) detrended fluctuation analysis scaling exponent alpha (DFA α) from the COP data. The 95% ellipse area, path length, and AP DFA α calculations are described elsewhere and represent typical spatiotemporal characteristics of balance [13,14]. The eEI metric was derived based on the EI used by the Equitest system. To simplify this process, the forward and backward sway angles were calculated as the inverse sine function of the anterior and posterior COP displacement, respectively, divided by an estimated COG height (56% of the participant height). The first trial of each condition served as a familiarization period, and only the final trial of each condition was used for analysis. Data that were outside of three times the SD from the mean of its experimental condition were removed from the analysis. In this manner, one trial each from SOT 3 and SOT 4 was removed, along with their VR condition pair.

### Statistical Analysis

To assess the relationship between EI and eEI, intraclass correlations of consistency and absolute agreement were calculated for similar conditions (Table 2). Intraclass correlation coefficient (ICC) values were interpreted as poor (<0.5), moderate (0.5-0.75), good (0.5-0.9), and excellent (>0.9) reliability [15]. Additionally, Pearson correlation coefficients were calculated to quantify the extent to which force plate measurements were associated with the EI calculated by the Equitest system within similar conditions. Correlation coefficients were interpreted as negligible (<0.3), weak (0.3-0.5), moderate (0.5-0.7), strong (0.7-0.9), or very strong (>0.9) relationships between pairs of variables [16].

**Table 2:** Summary of all testing conditions, their abbreviations, and the quality of visual, somatosensory, and vestibular information available in the condition.

Condition abbreviation	Equitest	Virtual reality	Information quality^a^
SOT^b^ 1	Eyes opened on a stable surface	Stable virtual surround on a stable surface	Vis^c^–Som^d^–Ves^e^
SOT 2	Eyes closed on a stable surface	Blacked out environment on a stable surface	Som–Ves
SOT 3	Eyes opened with a sway-referenced surround	Head-referenced virtual surround on a stable surface	*Vis*–Som–Ves
SOT 4	Eyes opened on a sway-referenced surface	Stable virtual surround on a foam surface	Vis–*Som*–Ves
SOT 5	Eyes closed on a sway-referenced surface	Blacked out environment on a foam surface	*Som*–Ves
SOT 6	Eyes opened with a sway-referenced surround and on a sway-referenced surface	Head-referenced virtual surround on a foam surface	*Vis*–*Som*–Ves

^a^In the column, normal text indicates accurate and italic text indicates inaccurate.

^b^SOT: sensory organization test.

^c^Vis: visual.

^d^Som: somatosensory.

^e^Ves: vestibular.

## Results

### Data Presentation and Assessment of the Raw Data

**Figure 2:**
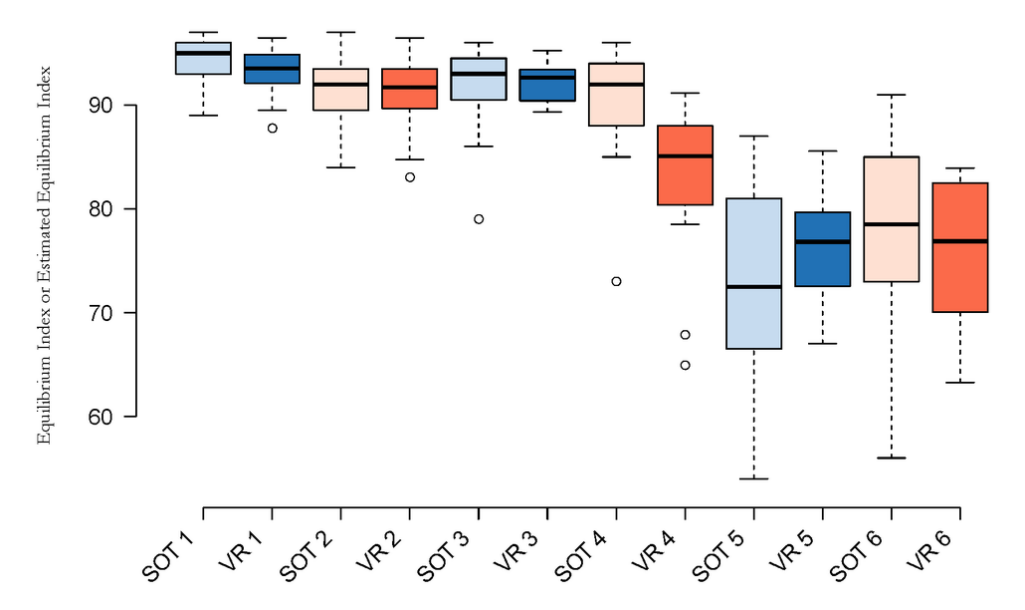
Boxplots of all data collected in comparable SOT (light) and VR (dark) conditions. The median value (thick line), IQR (box edges), and 95% CI (whiskers) are indicated. SOT: sensory organization test; VR: virtual reality.

Boxplots of the data showing the median (thick line), IQR (box edges), and 95% CI (whiskers) for each condition were created (Figure 2). Visual inspection of the data indicated symmetry in most conditions and increased variability in the more challenging conditions (conditions 4-6).

### Reliability of the eEI

**Figure 3:**
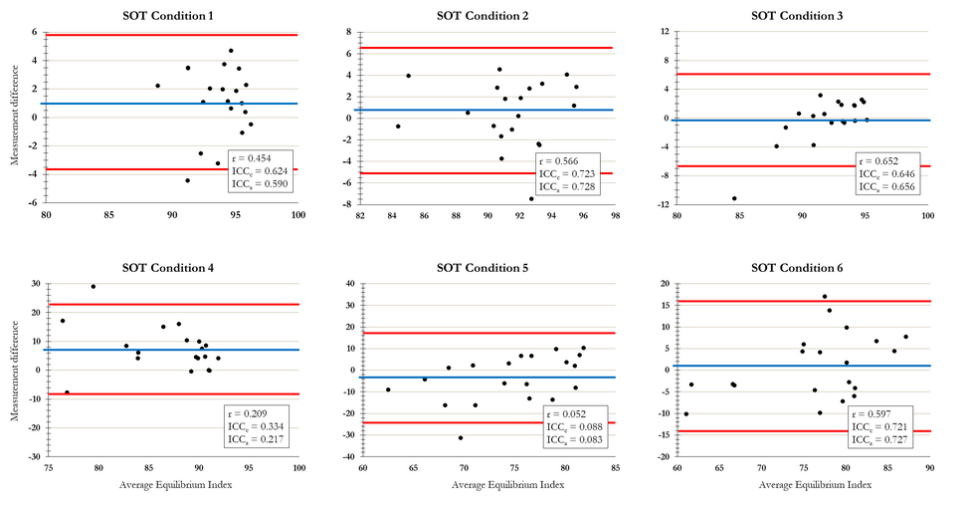
Bland-Altman plots comparing the equilibrium index and estimated equilibrium index from the Equitest and VR SOT, respectively. The Pearson correlation coefficient (r), intraclass correlation coefficient for absolute agreement (ICCa), and intraclass correlation coefficient for consistency (ICCc) are provided. SOT: sensory organization test; VR: virtual reality.

Intraclass correlations between EI and eEI in similar conditions were evaluated and are presented alongside Bland-Altman plots in Figure 3 [17]. SOT conditions 1, 2, 3, and 6 demonstrated moderate consistency and absolute agreement with their similar VR condition counterparts. Meanwhile, SOT conditions 4 and 5 showed poor consistency and absolute agreement with similar VR conditions. The Bland-Altman plots provide a visual representation of agreement between two measurements by plotting the absolute agreement or mean difference between measurements on the vertical axis against the average of the two measurements on the horizontal axis.

### Correlation of the EI With Force Plate Measurements

Pearson correlation coefficients were calculated between the Equitest EI and balance measures derived from COP data (Table 3). Weak to moderate significant correlations were identified between EI and eEI in SOT conditions 1 (*r*=0.454, *P*=.045), 2 (*r*=0.566, *P*=.009), 3 (*r*=0.652, *P*=.002), and 6 (*r*=0.597, *P*=.005). Additionally, weak to moderate significant correlations were identified between EI and 95% ellipse area in conditions 1 (*r*=−0.453, *P*=.045), 2 (*r*=−0.506, *P*=.02), and 6 (*r*=−0.500, *P*=.03) and AP DFA α in condition 1 (*r*=−0.511, *P*=.02). No other relevant correlations were identified between the Equitest EI and balance measurements derived from the COP data.

**Table 3:** Pearson correlation coefficients between force plate measurements (columns) and the equilibrium index during each sensory organization test condition.

Condition		eEI^a^	95% ellipse area	Path length	AP DFA α^b^
**SOT^c^ 1**					
	*r*	0.454	−0.453	−0.130	−0.511
	*P*	.045	.045	.59	.02
**SOT 2**					
	*r*	0.566	−0.506	−0.400	−0.041
	*P*	.009	.02	.08	.86
**SOT 3**					
	*r*	0.652	−0.329	−0.068	−0.234
	*P*	.002	.17	.78	.33
**SOT 4**					
	*r*	0.209	−0.143	−0.332	−0.007
	*P*	.39	.56	.16	.98
**SOT 5**					
	*r*	0.052	−0.242	−0.241	0.027
	*P*	.83	.30	.31	.91
**SOT 6**					
	*r*	0.597	−0.500	−0.334	−0.174
	*P*	.005	.03	.15	.46

^a^eEI: estimated equilibrium index.

^b^AP DFA α: anterior-posterior detrended fluctuation analysis scaling exponent alpha.

^c^SOT: sensory organization test.

## Discussion

This research has demonstrated the plausibility of using VR as an alternative to the Equitest when conducting a SOT. Although not a perfect replacement, eEI demonstrated reasonable correlations and ICCs with the clinical standard in several of the SOT conditions. Continued improvements to the VR testing environment need to be made to have more confidence in its use as a potential replacement. For example, the VR device may do a good job at mimicking the visual conditions of the SOT, but the foam mat might not equivocally disrupt somatosensory information compared with the SOT. This is supported by seeing higher correlations between EI and eEI in the intact than inaccurate somatosensory conditions (conditions 1, 2, and 3 versus conditions 4 and 5). Additionally, this study identified a number of correlations between the Equitest system and typical balance measurements derived from COP data on a force plate. Aside from eEI, 95% ellipse area and AP DFA α had some correlations with the clinical test. It is not surprising that these correlations were somewhat sparse as they distinctly measure different characteristics of balance. The SOT measures only AP sway magnitude, while COP data can be used to calculate sway magnitude in the frontal and sagittal planes combined or to measure aspects of how variability is structured in an individual plane. For example, 95% ellipse area quantifies the gross postural control behavior during quiet stance [18] and AP DFA α quantifies the structure of variability within an individual’s AP sway trajectory (ie, how random or deterministic the data is) [19], whereas EI evaluates how close an individual gets to a theoretical limit of stability [20]. The measures evaluated in this study were selected to represent a small array of postural control measurements, and future research should evaluate the clinical utility of individual metrics.

The recent surge in consumer-ready VR headsets has the potential to greatly reduce the cost of conducting balance assessments while also providing additional accessibility to sites outside of the clinic, for example, on the sideline during an athletic event. Likewise, using force plates opens access to raw, processed, and derived outcome measures that take advantage of the full scope of postural dynamics and present the opportunity to have more accurate information at the clinician’s disposal. In the future, it may even be possible to accurately assess balance (and gait) using only the self-contained tracking of VR headsets. This research serves as a point from which we can merge motor control assessments with the accelerating advancements in consumer technologies.

## Abbreviations

6DoF: six degrees of freedom
AP: anterior-posterior
COG: center of gravity
COP: center of pressure
DFA α: detrended fluctuation analysis scaling exponent alpha
eEI: estimated equilibrium index
EI: equilibrium index
ICC: intraclass correlation coefficient
SOT: sensory organization test
VR: virtual reality
